# Interband Absorption in Few-Layer Graphene Quantum Dots: Effect of Heavy Metals

**DOI:** 10.3390/ma11071217

**Published:** 2018-07-16

**Authors:** Ivan Shtepliuk, Rositsa Yakimova

**Affiliations:** 1 Department of Physics, Chemistry and Biology, Linköping University, SE-58183 Linköping, Sweden; rositsa.yakimova@liu.se; 2 Frantsevich Institute for Problems of Materials Science, NASU, 142 Kyiv, Ukraine

**Keywords:** DFT, few-layer graphene quantum dots, heavy metals, interaction, absorption spectroscopy, DOS

## Abstract

Monolayer, bilayer, and trilayer graphene quantum dots (GQDs) with different binding abilities to elemental heavy metals (HMs: Cd, Hg, and Pb) were designed, and their electronic and optical properties were investigated theoretically to understand deeply the optical response under heavy metal exposure. To gain insight into the nature of interband absorption, we performed density functional theory (DFT) and time-dependent density functional theory (TD-DFT) calculations for thickness-varying GQDs. We found that the interband absorption in GQDs can be efficiently tuned by controlling the thickness of GQDs to attain the desirable coloration of the interacting complex. We also show that the strength of the interaction between GQDs and Cd, Hg, and Pb is strongly dependent on the number of *sp*^2^-bonded layers. The results suggest that the thickness of GQDs plays an important role in governing the hybridization between locally-excited (LE) and charge-transfer (CT) states of the GQDs. Based on the partial density-of-states (DOS) analysis and in-depth knowledge of excited states, the mechanisms underlying the interband absorption are discussed. This study suggests that GQDs would show an improved sensing performance in the selective colorimetric detection of lead by the thickness control.

## 1. Introduction

Graphene quantum dots (GQDs) have gained considerable research interest over the past decade because of a possibility of their use for the optical detection of heavy metals by means of fluorescence spectroscopy, colorimetry, and ultraviolet–visible (UV-Vis) absorption spectroscopy [1,2,3,4,5,6,7,8,9,10,11,12,13,14,15,16,17,18]. The relevance of such studies is undoubtedly associated with the extremely high toxicity of bio-accumulated heavy metals for living creatures and, as a consequence, with a need to minimize the number of diseases and deaths caused by their negative impact [19,20,21]. The choice of the low-dimensional graphene-family materials as detection elements is justified by the unique properties of *sp*^2^-conjugated π-systems, which allow one to detect individual adsorbed atoms and molecules on their surface [22,23]. In contrast to the gapless two-dimensional (2D) graphene [24], zero-dimensional (0D) graphene quantum dots have an energy gap [25], which is strongly dependent on their size/shape [26], thickness [27], solvent (in most cases, GQDs are dispersed in organic electrolyte solutions) [28], edge termination (zigzag vs. armchair) [29], concentration of surface functional groups (oxygen-containing groups, such as epoxy, hydroxyl, and carboxylic acid groups) [30], and doping impurities (chlorine, nitrogen, boron, potassium, sodium, fluorine, sulfur, etc.) [31,32]. The existing detection methods are based on optical excitation of interband transitions in graphene quantum dots, with subsequent analytical processing of the output signal. In this regard, a deep understanding of the physical nature of such transitions is a primary objective for improving the efficiency of such sensing devices. It is important to note that the excitation of pristine, defect-free, and unmodified GQDs is mainly accompanied by electronic transitions between the doubly degenerate highest occupied and lowest unoccupied orbitals (HOMO and LUMO, respectively) [33]. The wave-functions of the electron and hole corresponding to the orbitals are completely delocalized over the *sp*^2^ plane [33]. The complexation between quantum dots and heavy metals leads to the activation of additional optical transitions involving hybridized orbitals (the carrier wave-function is distributed between the metallic adsorbate and the graphene quantum dots) and local states (the carrier wave-function is completely localized on the metallic adatom) [33]. The existence of such transitions manifests itself in the appearance of additional spectral bands at the absorption and fluorescence spectra or their quenching. In fact, the oscillator strength of the optical transition depends on the charge transfer between the analyst and the analyte. In the case of the weak physisorption of metals (controlled by the van der Waals interaction), the expected charge transfer is minimal, and the changes in the optical properties of the sensing material are believed to be negligible. In our previous work, optical transitions in monolayer quantum dots after binding with both neutral HM adatoms and divalent charged ions (Cd^2+^, Hg^2+^, and Pb^2+^) were investigated and possible ways of their detection were predicted [34]. Nevertheless, the experimental data suggest that the GQDs’ electrolyte solution may contain different fractions of flakes with different thicknesses, ranging from one monolayer (1ML) to three monolayers (3ML) [30]. This means that optical detection will be determined by the different types of interactions between the metal and quantum dots. Because of the thickness dependence of the HOMO-LUMO gap of the GQDs, interference of the overlapping absorption/emission spectral bands is expected. Therefore, the correct explanation of the detection mechanisms requires a fundamental understanding of the optical excitations both in single-layer and multi-layer graphene quantum dots. To our best knowledge, there are no systematic studies of the interband absorption in stacked GQDs interacting with heavy metals as a function of thickness.

It is important to note that most of the reports in this field are mainly devoted to the detection of divalent cations of heavy metals, namely Cd^2+^ [1], Hg^2+^ [9,10,11,12,13,14,15,16,17,18], and Pb^2+^ [2,3,4,5,6,7]. At the same time, being a toxic product that is released into the environment from coal combustion and oil burning heavy metals can exist in several chemical forms [35,36,37,38]: Oxidized form (HM^2+^), particulate-bound form (HM_p_), and elemental HM^0^. Although the oxidized and particulate-bound forms of heavy metals can be easily recognized and discriminated by using conventional detection facilities, it is still a challenge to detect elemental HM^0^ compared to other forms. This is mainly due to the poor solubility/reactivity of neutral HM atoms in water/electrolyte solutions [39,40,41] and, consequently, their weak binding ability to commonly-used sensing materials. Therefore, the present work mainly focuses on the deep investigation of electronic excitation processes in few-layer GQDs interacting with elemental heavy metals (Cd^0^, Hg^0^, and Pb^0^). For this aim, we performed density functional theory (DFT) and time-dependent DFT (TD-DFT) quantum-chemical calculations for the interacting complexes and predicted the transition density matrix (TDM) associated with the excited states. TDM analysis allowed us to identify the excited state character, and to understand the formation mechanism of the electron–hole pairs, including their delocalization and coherence length.

## 2. Theoretical Approach

**Figure 1:**
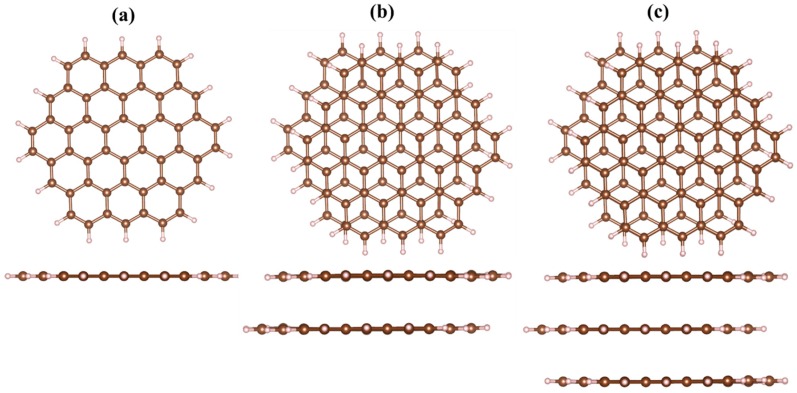
Optimized structures of zigzag-edged graphene quantum dots (GQDs) (top view and side view): (**a**) Monolayer, (**b**) bilayer, and (**c**) trilayer. Small balls correspond to hydrogen atoms; large atoms represent carbon species belonging to the GQDs.

All quantum chemistry calculations were performed using the Gaussian 09 Rev. D.01 program package [42]. To investigate the thickness effect on the excited states in the GQDs after complexation with elemental heavy metals (Cd^0^, Hg^0^, and Pb^0^), the monolayer (1ML), bilayer (2ML), and trilayer (3ML) GQDs were initially fully relaxed. As representative models of the few-layer GQDs, zigzag-edged C_54_H_18_ (circumcoronene), AB-stacked C_54_H_18_@C_54_H_18_, and ABA-stacked C_54_H_18_@C_54_H_18_@C_54_H_18_ structures were chosen (Figure 1). At the next stage, neutral heavy metal adatoms were located above the GQDs’ surface, and the complexed structures were fully optimized using the default convergence criteria (see the Figure S1, Supplementary Materials). We used the hybrid dispersion–corrected DFT functional M06-2X, which includes implicitly some modified parameters related to the Hartree-Fock exchange interaction [43]. It should be mentioned that the M06-2X function is suitable for the prediction of the weak van-der Waals interactions [44]. The 6-31G* basis set for carbon and hydrogen atoms, as well as a basis set developed by the Stuttgart-Dresden-Bonn group for the heavy metal atoms [45], were utilized to perform self-consistent calculations. The charge transfer was calculated using a Mulliken charge analysis.

Since the present work is mainly aimed to uncover the nature of the excited transitions in few-layer GQDs after complexation with elemental heavy metals, we calculated the ultraviolet-visible (UV-Vis) absorption spectra (including electronic transition energies and oscillator strengths) by using the time-dependent density functional theory (TD-DFT) approach at the same level of theory (M06-2X/6-31G*/SDD), implemented in the Gaussian 09 Rev. D.01 program. For such gas-phase calculations, we considered 12 excited electron states (transitions between occupied and unoccupied states). The oscillator strengths for each vertical transition in the GQDs determined the intensities of the absorption peaks.

To gain insight into the nature of the excited states, based on the results of the TD-DFT calculations we also predicted transition density matrices (TDM) for the lowest excitation states in the GQDs. The TDM analysis makes it possible to construct a two-dimensional representation of the distribution of the electron-hole, two-particle wave-functions. To be more exact, we estimated the probability of finding the electron and hole in the (q, r) atomic orbitals of all non-hydrogen atoms, x_i_ and y_i_, respectively:

(1) $${\left|\psi \left(x_{i},y_{i}\right)\right|}^{2}=\sum_{q\in x_{i}}\sum_{r\in y_{i}}{\left|\psi \left(q,r\right)\right|}^{2}$$

As has been shown previously, the resulting 2D colour-filled TDM maps can be useful for visualization of electron–hole coherence and the delocalization region, as was demonstrated for an assembly of conjugated carbon atoms upon excitation [46], and for understanding the excited state character (locally-excited state vs. charge-transfer state) [47].

It is noteworthy that any changes in the absorption spectra of GQDs in the visible range after complexation with heavy metals can correlate with colour changes perceived by the naked eye. In the light of a strong demand for equipment-free and sensitive detection of elemental heavy metals, it is, therefore, important to estimate the coloration of the GQDs before and after interaction with Cd, Hg, and Pb. This can be done under daylight conditions by using theoretically calculated absorption spectra and colour-matching functions. The methodology of the calculation of the perceived colour can be found elsewhere [48].

## 3. Results and Discussion

**Figure 2:**
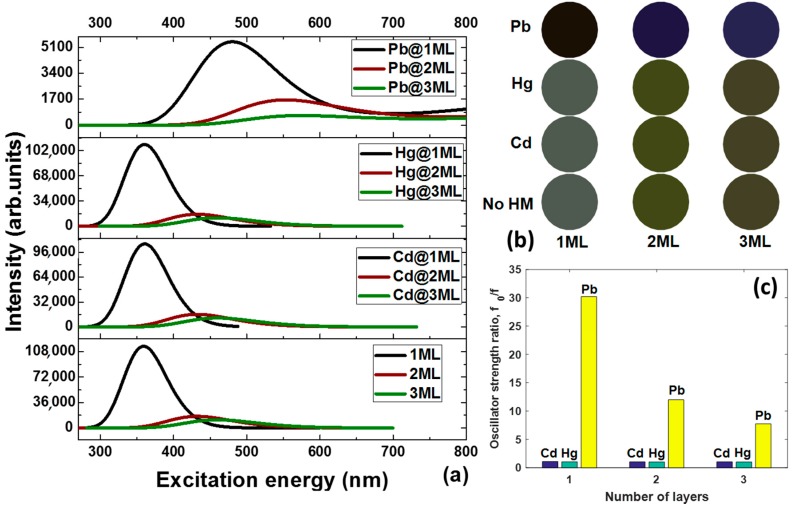
(**a**) Absorption spectra of the monolayer, bilayer, and trilayer GQDs interacting with Cd, Hg, and Pb, obtained from direct gas-phase time-dependent density functional theory (TD-DFT) calculations. (**b**) Palette of the perceived colours of thickness-varying GQDs before and after interaction with elemental heavy metals based on the predicted absorption spectra under daylight conditions. (**c**) The various oscillator strength ratios (*f*_0_/*f*) of the GQDs in the absence and presence of elemental metal adatoms, where *f*_0_ and *f* correspond to the oscillator strengths of the most probable optical excited transitions in the varying-thickness GQDs before and after binding with Cd, Hg, and Pb, respectively.

The optical properties of the graphene quantum dots interacting with elemental heavy metals depend on both their intrinsic parameters (the thickness in our case) and the binding energies of the metallic adsorbates. A study of these factors will shed light on how the thickness of GQDs influences the critical interaction strength, below which GQDs act like bulk stacked graphite-like structure or multi-layer carbon dots. We consider first the interband absorption in pristine GQDs as a function of the thickness. The calculated absorption spectra of GQDs of 1ML, 2ML, and 3ML thicknesses are presented in Figure 2a. A single sharp peak can be seen in each spectrum at 359, 431, and 459 nm for the monolayer, bilayer, and trilayer GQDs, respectively. Weak spectral features at 482 and 466 nm are also observed for the two latter structures. From this it is obvious that as the GQDs’ thickness increases, the absorption peak shifts to longer wavelengths. This can be explained by the energy gap shrinking due to the lift of the HOMO (highest occupied molecular orbital) level and lowering of the LUMO (lowest unoccupied molecular orbital) level in the few-layer GQDs. In addition, we noticed that the intensity of the absorption peak drastically decreases with an increasing thickness of the GQDs, and the absorption band line-width tends to become broader when moving from monolayer GQDs to trilayer GQDs. It is noteworthy that the red-shift of the absorption spectra in the visible range leads to changes in the perceived coloration of the GQDs from a grey (1ML GQDs) to mustard green colour (3ML GQDs). The corresponding simulated colours are displayed in Figure 2b.

**Figure 3:**
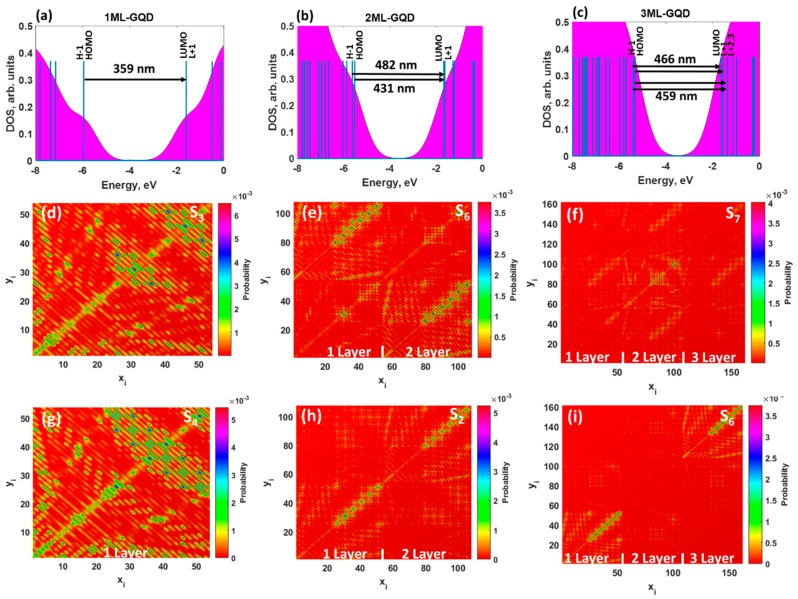
Density of states of the (**a**) monolayer, (**b**) bilayer, and (**c**) trilayer GQDs. Black arrows in (**a**–**c**) between the occupied and empty molecular orbitals represent electronic transitions (with highest values of the oscillator strengths) responsible for the optical absorption. Blue vertical lines denote the molecular orbitals. (**d**–**i**) Colour-filled maps of the transition density matrix of most probable (**d**) S_3_←S_0_ and (**g**) S_4_←S_0_ excited states in the monolayer GQDs; (**e**) S_6_←S_0_ and (**h**) S_2_←S_0_ in the bilayer GQDs; and (**f**) S_7_←S_0_ and (**i**) S_6_←S_0_ in the trilayer GQDs without interaction with elemental heavy metals. A colour scale bar is shown on the right of each contour plots.

To understand the origin of the absorption peaks, we performed DOS calculations for all considered structures. It was revealed that the H − 1 and HOMO, as well as L + 1 and LUMO, were degenerate π and π* orbitals in monolayer GQDs (Figure 3a). This is because they have the same energies, orbital compositions, and occupancy. Figure 3a also illustrates the transitions from the occupied states to the empty states. As one can see, the observed absorption spectrum of the 1ML GQDs is originating from the multi-level lowest single transitions between degenerated orbitals. In other words, the corresponding spectra are dominated by two different excited states: S_3_←S_0_ and S_4_←S_0_. The first excited state can be attributed to the HOMO→LUMO (49%) and H − 1→L + 1 (49%) transitions. Also, the other state is related to H − 1→LUMO (49%) and HOMO→L + 1 (49%) transitions. Molecular orbitals that are involved in these transitions are shown in Figure S2 (Supplementary Materials). The wave-functions of the aforementioned molecular orbitals were highly delocalized over the monolayer GQDs. Contrary to the doubly degenerate LUMO and HOMO in monolayer GQDs, the lowest orbitals in bilayer and trilayer GQDs split into two nondegenerate orbitals (see Figure 3b,c, see also Figures S3 and S4, Supplementary Materials). Such a splitting is responsible for the change in the nature of the electronic excitations: From the doubly degenerate excited state in the monolayer to a set of excited states in the 2ML and 3ML GQDs. The absorption spectrum of bilayer GQDs originates from the excited state, with oscillator strength of 0.3693: H − 1→LUMO (46%) and HOMO→L + 1 (41%) transitions. We find also a weak contribution of the electronic excitation S_3_←S_0_ at 482 nm, with oscillator strength of as small as 0.0273, to the absorption spectrum of the AB-stacked GQDs.

From the analysis of the electronic nature of the exited states in the trilayer GQDs, one can conclude that the dominant contribution to the interband absorption arises from H − 1→LUMO (31%) and HOMO→L + 1 (54%) transitions. Tables S1–S3 (Supplementary Materials) summarize the electronic transitions, which contribute to the absorption spectra of the thickness-varying GQDs. It is important to note that the wave-function of the molecular orbitals, which are involved in the optical transitions in stacked GQDs, is unequally shared between the two and three layers in the AB and ABA configurations (see Figures S3 and S4, Supplementary Materials). As a consequence of the nonuniform distribution of the wave-function, a drastic reduction of the oscillator strength of the allowed transitions in the stacked GQDs was observed compared to the monolayer GQDs.

To shed more light on the nature of the interband absorption in thickness-varying GQDs, we computed the transition density matrix for the two lowest excited states in each GQD (Figure 3d–i). It should be mentioned that, in most cases, the excited state could have two components: A locally-excited (LE) state and a charge-transfer (CT) state [49]. A detailed analysis of the matrix elements allows us two distinguish between the two of them. Indeed, the diagonal terms of the transition density matrix correspond to the charge variation of the corresponding atoms and, as a consequence, represent the LE character of the excited state, while the off-diagonal terms are related to the CT component, exhibiting the electron-hole coherence between the corresponding atoms. The 2D-grid maps for the monolayer GQDs (Figure 3d,g) indicate that the LE character dominated both the S_3_ and S_4_ states. This is due to the fact that the graphene quantum dots are highly delocalized systems and, thus, a strong coherence between *sp*^2^-bonded carbon atoms was observed. It is interesting to note that both S_0_→S_3_ and S_0_→S_4_ transitions preferentially involve “edge” carbon atoms labelled 25–54, while centered carbon atoms (labelled 1–24) belonging to inner hexagonal rings are less involved in excited transitions. In the case of bilayer graphene quantum dots (Figure 3e,h), the contribution of the CT component to the lowest excited state, namely S_6_, becomes larger compared to that of 1ML GQDs, and the symmetric (mirror) brightest regions within the corresponding map are found in the upper left corner and in the bottom right corner. This means that the CT component is localized mainly on the edge carbon atoms in the first layer and second layer of GQDs, respectively. The presence of the second layer causes a larger spatial separation of the hole and electron wave-functions and, as a result, a small wave-function overlap integral and a reduced oscillator strength of the corresponding transition. When the third layer is added to the GQDs, the brightest zone is concentrated in the center of the colour-filled map of the trilayer GQDs (Figure 3f,i), implying a preferential participation of the inner carbon atoms of the second layer in the optical transitions. Furthermore, the corresponding matrix elements are diagonal, indicating the partial LE character of the S_7_ state. We also noticed the presence of the CT component, originating from inner hexagonal rings in each layer. Despite the negligible low values of the oscillator strengths for S_2_ and S_6_ transitions in the bilayer and trilayer GQDs (see Figure 3h,i), the brightest regions for both of them are found along the diagonal, confirming the dominant role of locally-excited states. Additional evidence for the correct determination of the type of electron excitation can be found through comparing the charge-transfer length (∆*r*), the electron-hole wave-function overlap integral (*S*), and the distance between centroids of the hole and electron (*D*) for the corresponding excited state [50]. All these parameters are listed in Tables S4–S6 (Supplementary Materials). The Δ*r* and *D* of excited states in pristine monolayer and bilayer GQDs are negligibly small, while these states are characterized by a large overlap integral. It means that the electrons and holes are localized without an obvious charge transfer. Moving from the monolayer GQDs to the trilayer GQDs, we noticed an increase of the CT component contribution to the corresponding excited states, which is confirmed by the increase of the parameters, Δ*r* and *D*, and a decrease of the overlap integral (Table S6). Therefore, one can classify these states as LE-CT hybridized states.

We turn now to present the results obtained for the heavy metals adsorption on the thickness-varying GQDs. We have found optimized adsorption configurations that represent the interaction of elemental Cd, Hg, and Pb with GQDs. A schematic representation of the optimized geometries is displayed in Figure S1 (Supplementary Materials). Our calculations predict that the number of layers in GQDs does not influence the preferential adsorption sites for all considered metals (the hollow site for Cd and Hg, and the bridge site for Pb). The calculations also confirm that the second and third layer affect the binding energy of the heavy metals, though in a manner opposite to the monolayer GQDs. In Dataset S1 (Supplementary Materials), we summarize the most important parameters describing the different configurations. Since the charge-transfer contribution to the total interaction energy decreases for thicker GQDs, it is reasonable to assume that the interlayer van-der-Waals dispersive forces (attractive in nature) between layers in GQDs somehow enhance the binding energy of Cd and Hg. A similar clarification was proposed by Hardcastle et al. [51] to explain the high binding energy of Au, Cr, and Al atoms adsorbed on few-layer graphene. Unlike the adsorption of Cd and Hg, the binding energy of Pb decreases when the thickness increases, indicating the involvement of other forces in the binding mechanism. In this case, we cannot ascribe this decrease only to the vanishing charge transfer term. It is obvious that the repulsive forces also contribute to the total interaction energy. To rationalize the observed phenomena, we also calculated the dipole moments of graphene quantum dots before and after interaction with heavy metals (Dataset S1, Supplementary Materials). Adsorption of metal species onto GQDs unbalances the electron density at the interface, thereby causing a charge separation and a resulting dipole moment. For all metals, the complexation with GQDs modifies only the *z*-component of the dipole moment (which is perpendicular to the plane of the GQDs), while the *x* and *y* components are still negligibly small (almost zero). In our case, the dipole’s direction points from the negatively charged GQDs to the positively charged metal adsorbates. While pristine GQDs exhibit a zero electric dipole moment when they interact with heavy metals, the resulting dipole moments of 0.96 Debye, 0.70 Debye, and 1.34 Debye are obtained for Cd, Hg, and Pb adsorbates, respectively. The *z*-component of the electric dipole moment was found to increase by 71% and 88% after cadmium and mercury adsorption as the number of layers in the GQDs increases from monolayer to trilayer. It is apparent that more *sp*^2^-bonded carbon layers gives rise to stronger polarization of the Cd and Hg adsorbates, and a larger value of the dipole moment. Our calculations led to the finding of another unexpected feature. The dispersive forces between layers in Pb GQDs do not only lead to stronger adsorption—but also to the formation of complexes with a smaller interaction strength. The dipole moment of the GQDs complexed with lead adatoms tends to become smaller when the number of layers increases. As shown in Dataset S1 (Supplementary Materials), the adsorption of Pb reduces the dipole moment of GQDs by 26% from 1.34 Debye (for monolayer) to 0.99 Debye (for trilayer). The decrease of the dipole moment of the Pb GQDs complexes when the number of layers increases can be caused by a partial screening of the dipole moments, which is governed by a combined effect originating from the increase of repulsive forces and decrease of the charge-transfer contribution.

Then, we extended our investigations to the interband absorption in thickness-varying GQDs interacting with heavy metals. From Dataset S1 (Supplementary Materials), it is clearly seen that the HOMO-LUMO gap of GQDs complexed with elemental heavy metals seems to follow the trend of the pristine GQDs, which is reduced when the number of *sp*^2^-bonded carbon layers increases. Our calculations demonstrate that there is a minimal decrease in LUMO energies for all interacting complexes compared to the pristine GQDs, but the HOMO energies change more dramatically. Furthermore, in contrast to the weakly-bonded complexes with Cd and Hg, the complexation between GQDs and Pb causes a more drastic increase in the HOMO energy. The up-shifted HOMO energies give rise to a narrowing of the HOMO-LUMO gap. The phenomenon of the HOMO-LUMO gap narrowing, due to both adsorbates’ effect and thickness influence, was also verified by the red-shift in the absorption spectrum (see Figure 2a) and corresponding coloration changes (Figure 2b). For all systems, we observe a quenching of the absorption intensity and a red-shift due to the thickness effect. Comparing the spectra of the GQDs before and after interaction with heavy metals, we note that the absorption spectra are only slightly affected by the Cd and Hg adatoms, maintaining almost the same spectral shape. The quenching and red-shift of the absorption spectra caused by Cd and Hg adsorption determine changes of the perceived grey component to a mustard coloration, which is in good agreement with the thickness-dependent coloration of the pristine GQDs. Contrastingly, the change in spectra affected by the presence of lead is significant, especially the position of the adsorption bands, which is strongly red-shifted by the Pb effect. The corresponding colours are thus turned from black to dark blue. It is interesting to note that, due to the increase in the binding energy of Pb with an increasing thickness of the GQDs, the oscillator strength ratio for the resulting complexes tends to become smaller (Figure 2c), implying that the few-layer GQDs are less sensitive to elemental Pb in comparison to monolayer GQDs.

**Figure 4:**
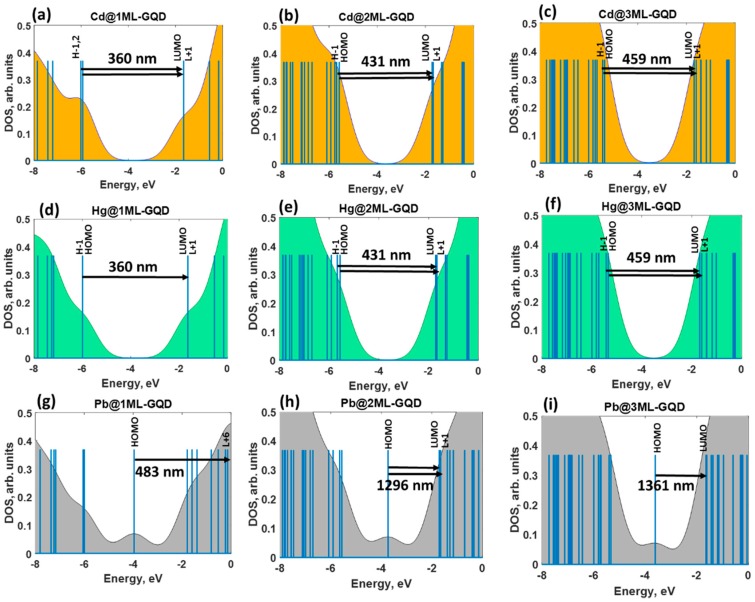
Calculated density of the states (shaded area) of the thickness-varying GQDs interacting with elemental heavy metals: (**a**–**c**) Cd, (**d**–**f**) Hg, and Pb (**g**–**i**), respectively. Blue vertical lines correspond to the molecular orbitals. Horizontal black arrowed lines show the excited transitions between the available occupied and unoccupied electronic levels.

**Figure 5:**
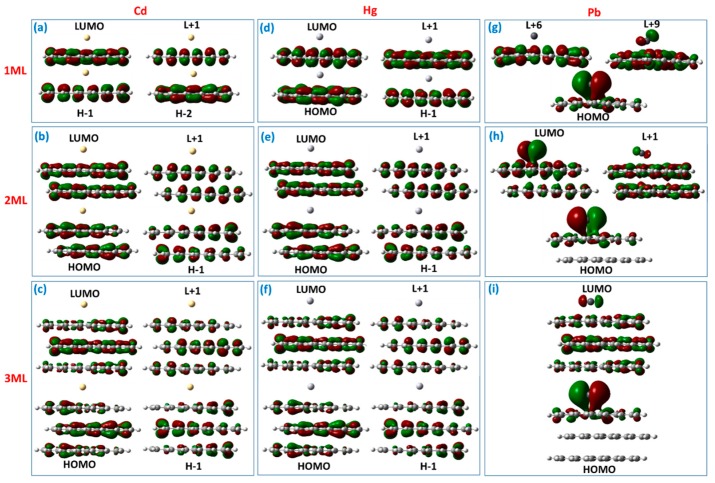
Images demonstrating the spatial distribution of wave-functions corresponding to occupied and unoccupied orbitals, which are involved in electronic transitions in monolayer, bilayer, and trilayer GQDs after interaction with elemental heavy metals: (**a**–**c**) Cd, (**d**–**f**) Hg, and (**g**–**i**) Pb, respectively. The red and green colours indicate positive and negative phases in the wave function, respectively. The orbitals are drawn at an isosurface value of 0.02.

The combined analysis of the DOS spectra and orbital composition of the molecular levels involved in excited transitions allowed an understanding the nature of the optical response of the thickness-varying graphene quantum dots under heavy metals exposure. From Figure 4a–f, it is clearly seen that the absorption spectra of GQDs interacting with Cd and Hg are dominated by the same optical transitions at 360 nm (monolayer), 431 nm (bilayer), and 459 nm (trilayer), respectively. The full assignments of the corresponding transitions are summarized for each absorption band in Dataset S2 (Supplementary Materials). In fact, despite the van-der-Waals interaction between these two metals and GQDs, the corresponding molecular orbitals are completely delocalized over the GQDs (Figure 5a–f).

**Figure 6:**
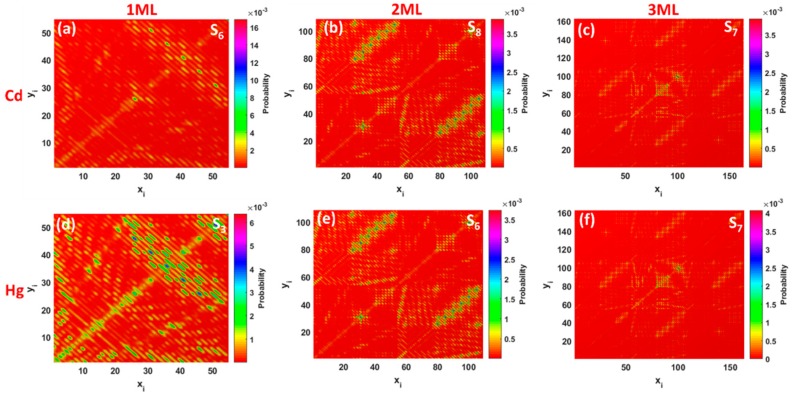
Two-dimensional representations of the simulated transition density matrix corresponding to the lowest excited states in thickness-varying GQDs after interaction with elemental heavy metals: (**a**–**c**) Cd and (**d**–**f**) Hg on monolayer (1ML), bilayer (2ML), and trilayer (3ML) GQDs, respectively. Colour bars are given on the right.

As was mentioned before, each excited state is related to the transition density matrix, which gives a clear representation of the degree of electron-hole coherence after photon absorption. Note that the electron–hole coherence associated with the most probable electronic transitions, namely S_6_ and S_3_, occurring in monolayer GQDs complexed with Cd and Hg are mainly delocalized on the whole interacting complex, indicating the LE character of these states (Figure 6a,d). The small values of the Δ*r* index and distance between centroids of the electrons and holes, as well as the large value of the electron-hole overlap integral, also confirm that the electron and holes belong to the same group of carbon atoms (Dataset S3, Supplementary Materials). On the other hand, the increase in the number of layers in GQDs causes a small interlayer charge transfer. This is evidenced by the increase of the parameters, Δ*r* and *D*, and the decrease of the overlap integral for the main excited states in bilayer and trilayer GQDs after interaction with elemental Cd and Hg species (Dataset S3, Supplementary Materials). TDM representations for S_8_, S_6_, and S_7_ states (Figure 6b,c,e,f) indicate that the corresponding electron−hole pairs are localized both along the diagonal elements and the off-diagonal elements. As a result, a mixture of intralayer local excitation within the respective layers coupled with interlayer CT between the different layers occurs.

**Figure 7:**
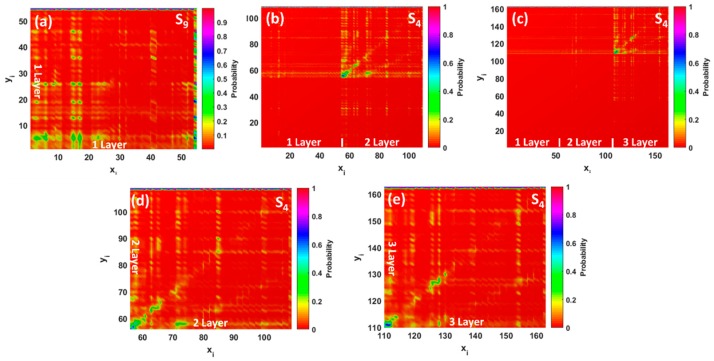
Two-dimensional representations of the simulated transition density matrix corresponding to the lowest excited states in thickness-varying GQDs after interaction with Pb: (**a**) Pb on monolayer (1ML), (**b**) Pb on bilayer (2ML), and (**c**) Pb on trilayer (3ML) GQDs, respectively. (**d**,**e**) demonstrate zoomed transition density matrix (TDM) patterns for S_4_ excited states in bilayer and trilayer GQDs interacting with Pb. Since the oscillator strengths of the main electronic transitions in Pb GQDs are small, the corresponding TDM patterns were normalized by the maximal value of the probability of finding the generated electron-hole pairs. Colour bars are given on the right.

For the GQDs interacting with Pb species, we observed the dominating absorption bands at 483, 1296, and 1361 nm for monolayer, bilayer, and trilayer GQDs, respectively (Figure 4g–i). These low-intensity absorption features correspond to the S_9_, S_4_, and S_4_ excited states, with rather small oscillator strengths. The electron distributions of some frontier molecular orbitals involved in the absorption spectra of the Pb GQDs complexes are shown in Figure 5g–i. The major absorption band at 483 nm originates from the combination of the HOMO→L + 6 (65%) and HOMO→L + 9 (14%) transitions. Molecular orbital analysis confirmed that the HOMO is shared between Pb and GQDs (contribution of Pb is about 79%), while the L + 6 is a less hybridized orbital with a small contribution from Pb (only 8%). This is contrary to the L + 9, which was delocalized over the plane of the GQDs. The transition density matrix describing the S9 state belongs to a CT-type excitation (Figure 7a). It is shown by the simultaneous presence of the electron-hole coherence in both diagonal and off-diagonal directions (bright regions in the bottom right corner of the map as well as off-diagonal matrix elements corresponding to the charge transfer between carbon atoms and Pb). Furthermore, the parameters, Δ*r* and *D*, were estimated to be about 2.15 and 2.02 Å (see Dataset S3, Supplementary Materials), while the overlap integral was very small (0.20). Contrary to the monolayer GQDs, interaction of the bilayer and trilayer GQDs with elemental Pb causes a red-shift of the absorption wavelengths and a subsequent reduction of the absorption intensity. In principle, the nature of the observed low-intensity absorption features is very similar to the monolayer case ( Figure 4h,i and Figure 5h,i ), including the electronic transitions between occupied orbitals (that related to the strongly hybridized HOMO) and the lowest unoccupied energy levels (weakly hybridized LUMO and L + 1). In both cases, the wave function of the HOMO level is shared by the lead adsorbate and the topmost layer in stacked GQDs, while LUMO and L + 1 are mainly delocalized over the whole GQDs, with a small contribution from lead. According to the TDM representation for the corresponding excited states (S_4_ states in both cases), the electron–hole coherence associated with the optical transitions at 1296 and 1361 nm with negligible small oscillator strengths is mainly delocalized on the topmost layer (second layer in bilayer GQDs and third layer in trilayer GQDs), demonstrating the intralayer charge transfer features and charge transfer between carbon atoms and lead adsorbates (Figure 7b–e). In this regard, we revealed a significant increase of the parameters, Δ*r* and *D*, in comparison to monolayer GQDs (see Dataset S3, Supplementary Materials), which confirms the intramolecular CT character of S_4_ excited states in few-layer GQDs after interaction with Pb.

## 4. Conclusions

In this work, we have designed stacked GQDs with a different number of layers (1–3), and theoretically investigated the effect of adsorbed heavy metals (Cd, Hg, and Pb) on the interband absorption. To find out the correlation between the interaction strength, coloration changes, electronic, and optical properties, we performed complexed DFT and TDDFT calculations and revealed that an increase of the GQDs’ thickness strengthens the interaction between GQDs and Cd (Hg) due to the effect of the dispersive forces and weakens the interaction with Pb, followed by the reduction of the charge transfer and the dipole moment. In addition, the interaction strength modulation reduced significantly the absorption of visible-light in the GQDs complexed with Pb, which originates from the red-shifted absorption due to the HOMO-LUMO gap shrinking and a large charge transfer from elemental lead to the GQDs. This is beneficial for the selective colorimetric detection of lead because of the large quenching of the optical signal. Moreover, there was a complicated relationship between the electron-hole coherence associated with the excited states in few-layer GQDs and the orbital composition of the molecular level involved in the corresponding transitions. When increasing the number of *sp*^2^-bonded layers in GQDs before and after interaction with Cd and Hg, we observed a transformation of the excited state character from the pure locally-excited (LE) state to the LE-CT hybridized state, which can be ascribed to the redistribution of the molecular energy orbitals between different layers. Through the analysis of the transition density matrices, we found strong charge-transfer channels between monolayer GQDs and Pb, as well as an increase of the charge-transfer length, with increasing of the number of layers in the GQDs. Finally, our work shows that thickness-varying GQDs would be promising materials for the preparation of high-performance and printable sensor arrays, with substantial chemical selectivity for the identification and quantification of elemental lead. Large optical signal quenching observed under Pb exposure may provide a unique colour change profile that is a fingerprint for the elemental lead.

## Supplementary Materials

The following are available online at http://www.mdpi.com/1996-1944/11/7/1217/s1, Figure S1: Optimized structures of the thickness-varying GQDs interacting with Cd (a–c), Hg (d–f) and Pb (g–i); Figures S2–S4:Images demonstrating the spatial distribution of wave-functions corresponding to occupied and unoccupied orbitals, which are involved in electronic transitions in monolayer, bilayer and trilayer GQDs; Table S1: Electronic transitions in 1ML-GQDs, Table S2: Electronic transitions in 2ML-GQDs, Table S3: Electronic transitions in 3ML-GQDs; Table S4: Electronic transitions in 1ML-GQDs: TDM analysis, Table S5: Electronic transitions in 2ML-GQDs: TDM analysis, Table S6: Electronic transitions in 3ML-GQDs: TDM analysis; Dataset S1: Parameters of the GQDs after complexation with HMs, Dataset S2: Electronic transitions in HMs@GQDs, Dataset S3: Electronic transitions in HMs@GQDs: TDM analysis.
