# Supplementary material for: Interband Absorption in Few-Layer Graphene Quantum Dots: Effect of Heavy Metals
Source: Materials (Basel). 2018 Jul 16;11(7):1217. doi: 10.3390/ma11071217 (PMC6073920; doi:10.3390/ma11071217)
Supplement: Supplementary file 1 [file materials-11-01217-s001.pdf]

materials

Interband Absorption in Few-Layer Graphene Quantum Dots: Effect of Heavy Metals

Ivan Shtepliuk [1,2]* and Rositsa Yakimova [1]

Supplementary Materials:

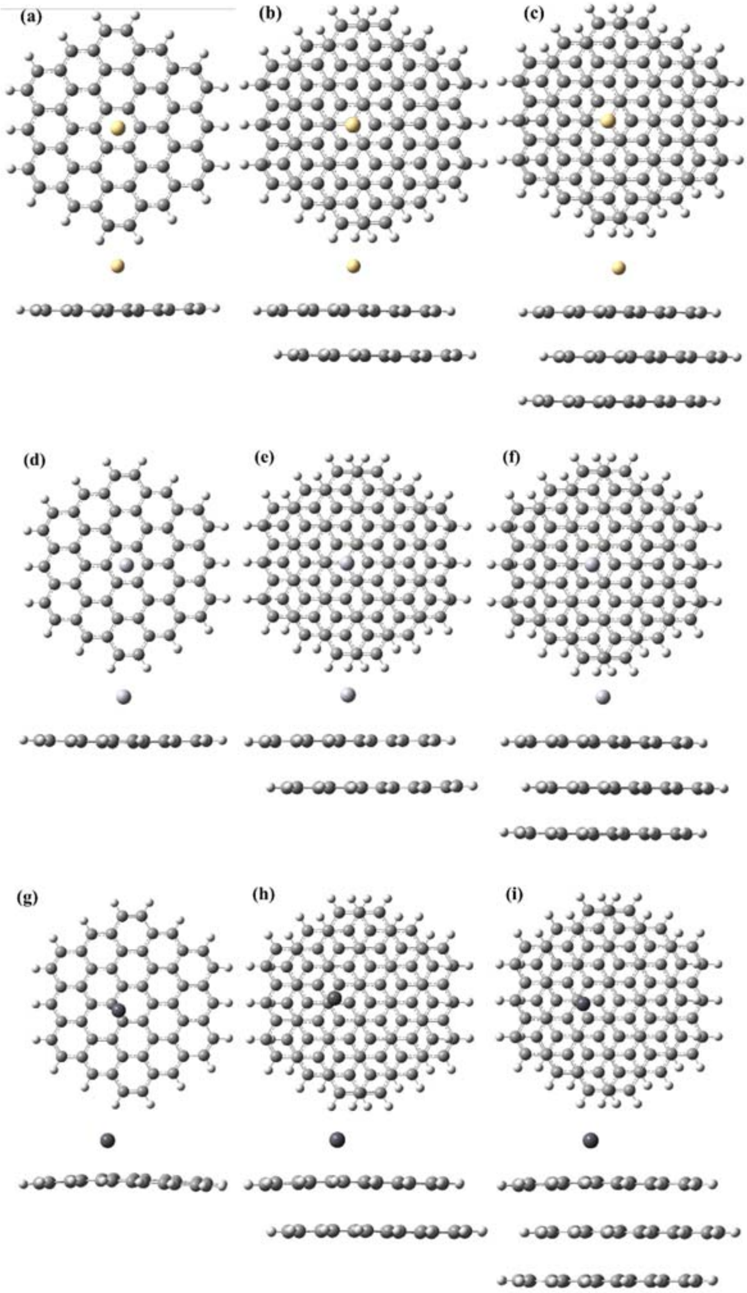

Figure S1. Optimized structures of the thickness-varying GQDs interacting with Cd (a-c), Hg (d-f) and Pb (g-i)

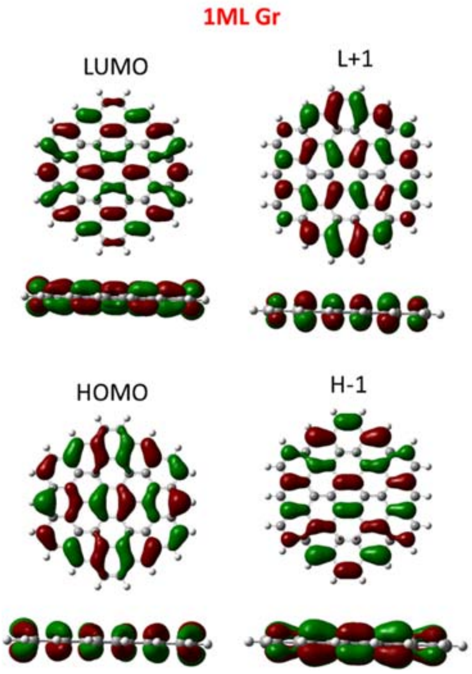

Figure S2. Images demonstrating the spatial distribution of wave-functions corresponding to occupied and unoccupied orbitals, which are involved in electronic transitions in monolayer GQDs. The red and green colours indicate positive and negative phases in the wave function, respectively. The orbitals are drawn at an isosurface value of 0.02.

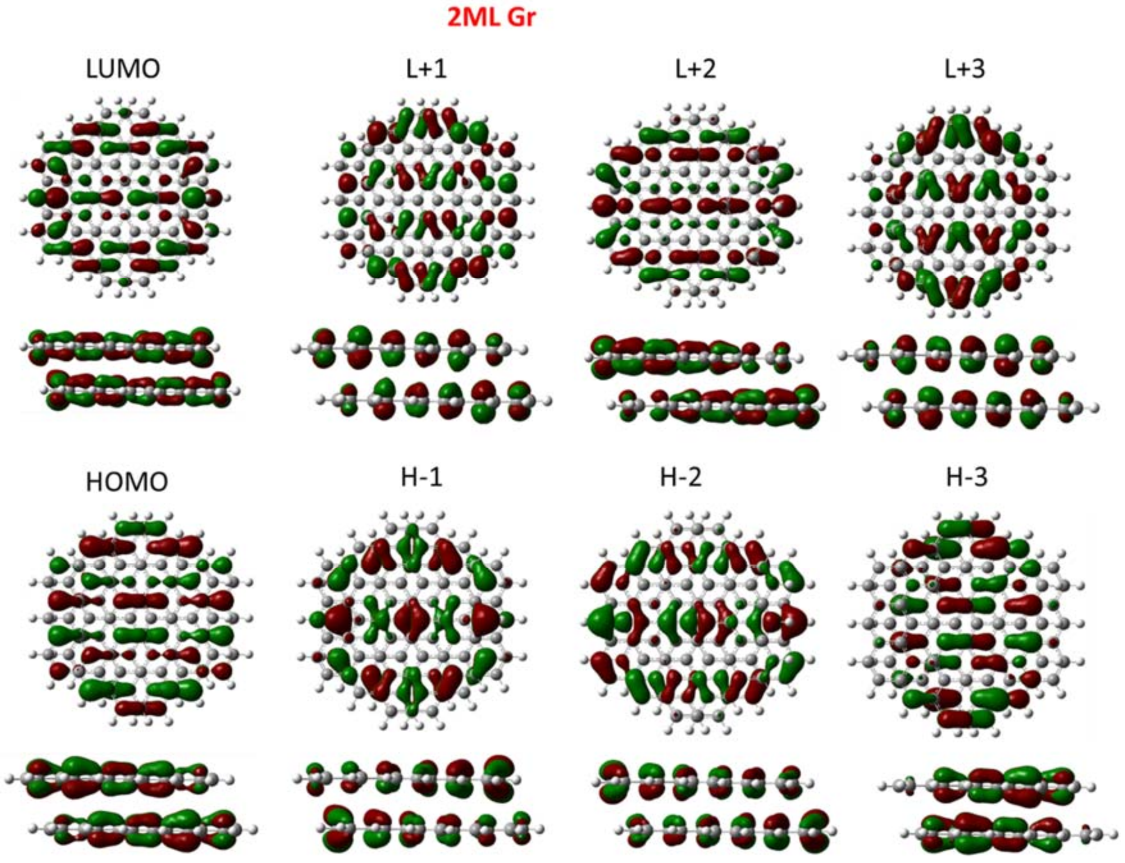

Figure S3. Images demonstrating the spatial distribution of wave-functions corresponding to occupied and unoccupied orbitals, which are involved in electronic transitions in bilayer GQDs. The red and green colours indicate positive and negative phases in the wave function, respectively. The orbitals are drawn at an isosurface value of 0.02.

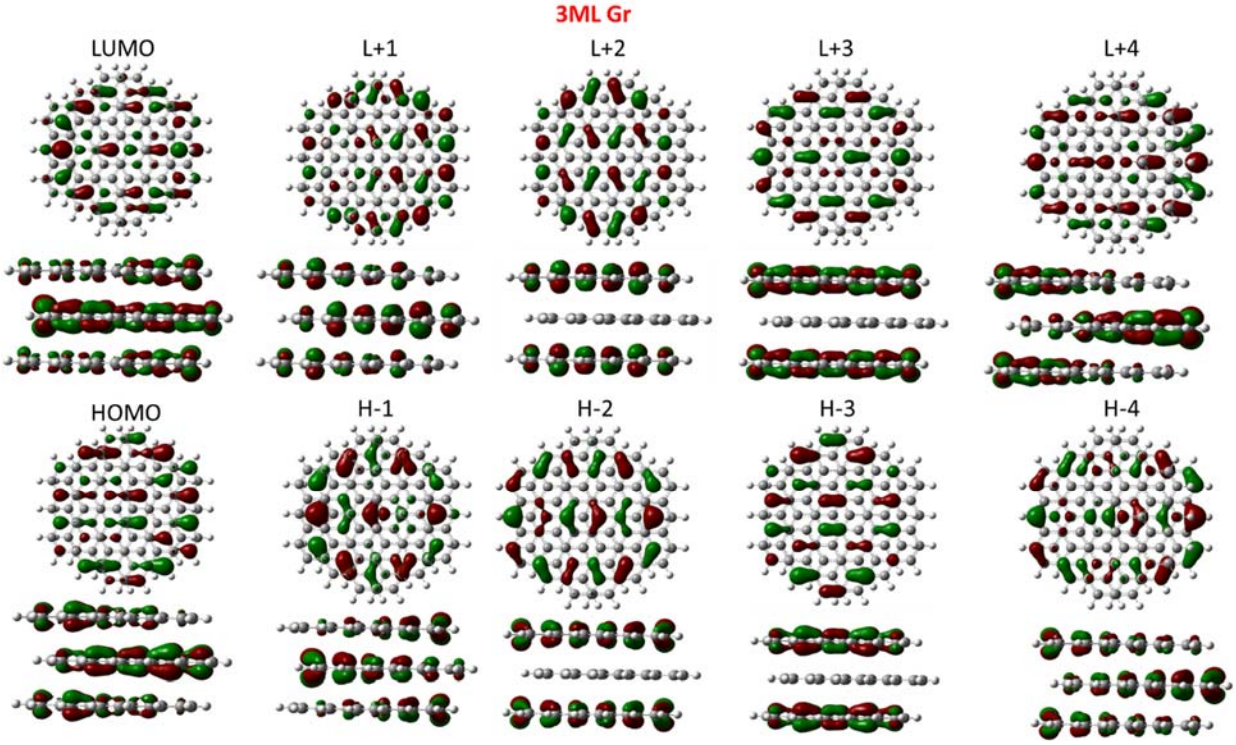

Figure S4. Images demonstrating the spatial distribution of wave-functions corresponding to occupied and unoccupied orbitals, which are involved in electronic transitions in trilayer GQDs. The red and green colours indicate positive and negative phases in the wave function, respectively. The orbitals are drawn at an isosurface value of 0.02.

Table S1. Electronic transitions in 1ML-GQDs

No.	Wavelength (nm)	Osc. Strength	Major Contribs
3	359.07	1.4285	H-1->L+1 (49%), HOMO->LUMO (49%)
4	359.07	1.4287	H-1->LUMO (49%), HOMO->L+1 (49%)

Table S2. Electronic transitions in 2ML-GQDs

No.	Wavelength (nm)	Osc. Strength	Major Contribs	Minor Contribs
2	482.82	0.0273	H-1->LUMO (47%), HOMO->L+1 (48%)	H-2->L+2 (2%)
6	431.92	0.3693	H-1->LUMO (46%), HOMO->L+1 (41%)	H-3->L+3 (3%), H-2->L+2 (8%)
8	412.79	0.0027	H-3->LUMO (45%), H-1->L+3 (12%), HOMO->L+2 (35%)	H-2->L+1 (3%)
9	406.77	0.0249	H-2->L+1 (46%), H-1->L+3 (36%), HOMO->L+2 (12%)	H-3->LUMO (4%)

Table S3. Electronic transitions in 3ML-GQDs

No.	Wavelength (nm)	Osc. Strength	Major Contribs	Minor Contribs
1	543.09	0.0003	H-1->L+1 (18%), HOMO->LUMO (70%)	H-3->L+3 (2%)
4	496.39	0.0037	H-1->L+1 (73%), HOMO->LUMO (22%)	
6	466.86	0.0468	H-3->L+1 (10%), H-2->LUMO (19%), H-1->L+3 (21%), HOMO->L+2 (42%)	H-4->L+3 (3%)
7	459.745	0.2135	H-1->LUMO (31%), HOMO->L+1 (54%)	H-4->LUMO (5%), H-4->L+4 (4%), H-2->L+3 (4%)
8	445.63	0.0216	H-4->LUMO (51%), H-2->L+3 (11%), H-1->L+4 (10%)	H-5->L+1 (3%), H-3->L+2 (9%), H-1->LUMO (2%), HOMO->L+5 (8%)
10	440.44	0.0005	H-1->L+3 (42%), HOMO->L+2 (44%)	H-5->L+2 (3%), H-2->LUMO (8%)

11	432.03	0.0004	H-2->LUMO (61%), H-1->L+3 (25%)	H-4->L+3 (6%), HOMO->L+2 (4%)

Table S4. Electronic transitions in 1ML-GQDs: TDM analysis

No.	Wavelength (nm)	Δr (A)	Integral of Overlap of Hole-electron (S)	Distance between Centroid of Hole and Electron (D, A)	Property
3	359.07	0.000014	0.7963548	0.000014	LE
4	359.07	0.000014	0.7963874	0.000014	LE

Table S5. Electronic transitions in 2ML-GQDs: TDM analysis

No.	Wavelength (nm)	Δr (A)	Integral of Overlap of Hole-electron (S)	Distance between Centroid of Hole and Electron (D, A)	Property
2	482.82	0.000404	0.7626221	0.000117	LE
6	431.92	0.000403	0.7766723	0.000128	LE
8	412.79	0.000531	0.5453574	0.000475	LE
9	406.77	0.000814	0.5680931	0.000343	LE

Table S6. Electronic transitions in 3ML-GQDs: TDM analysis

No.	Wavelength (nm)	Δr (A)	Integral of Overlap of Hole-Electron (S)	Distance between Centroid of Hole and Electron (D, A)	Property
1	543.09	0.020755	0.5614517	0.000505	CT-LE
4	496.39	0.041651	0.5562094	0.038468	CT-LE
6	466.86	0.761299	0.6872514	0.354292	CT-LE
7	459.745	0.064056	0.7225038	0.142090	CT-LE
8	445.63	0.150939	0.6701094	0.077652	CT-LE
10	440.44	0.802831	0.4384059	0.698093	CT-LE
11	432.03	0.679109	0.6491757	0.051356	CT-LE

Dataset S1. Parameters of the GQDs after complexation with HMs

Parameters of the GQDs after complexation with Cd

GQDs	Total Energy, Hartree	HOMO Energy, Hartree	LUMO Energy, Hartree	HOMO-LUMO Gap, eV	Charge on Atom	Binding Energy, eV	Dipole Moment, Debye
1ML	-2224.43233953	-0.21315	-0.06430	4.050	0.106	0.410	0.9665
2ML	-4281.30035473	-0.20470	-0.06380	3.834	0.096	0.422	1.4985
3ML	-6338.16421714	-0.19898	-0.06028	3.774	0.094	0.429	1.6559

Parameters of the GQDs after complexation with Hg

GQDs	Total Energy, Hartree	HOMO Energy, Hartree	LUMO Energy, Hartree	HOMO-LUMO Gap, eV	Charge on Atom	Binding Energy, eV	Dipole Moment, Debye
1ML	-2210.16029749	-0.22671	-0.06381	4.432	0.133	0.4049	0.7026
2ML	-4267.02814769	-0.20830	-0.06338	3.943	0.122	0.4109	1.1791
3ML	-6323.89256862	-0.19861	-0.05993	3.773	0.119	0.4328	1.3228

Parameters of the GQDs after complexation with Pb

GQDs	Total Energy, Hartree	HOMO Energy, Hartree	LUMO Energy, Hartree	HOMO-LUMO Gap, eV	Charge on Atom	Binding Energy, eV	Dipole Moment, Debye
1ML	-2060.10407484	-0.14716	-0.06781	2.159	0.314	0.5383	1.3473
2ML	-4116.97106080	-0.13598	-0.06253	1.998	0.267	0.5208	1.2595
3ML	-6173.83496799	-0.13008	-0.05790	1.964	0.238	0.5288	0.9913

Dataset S2. Electronic transitions in HMs@GQDs

Electronic transitions in Cd^0@1ML-GQDs

No.	Wavelength (nm)	Osc. Strength	Major Contribs	Minor Contribs
5	360.54	1.3168	H-2->LUMO (43%), H-1->L+1 (43%)	H-2->L+1 (5%), H-1->LUMO (5%)
6	360.54	1.3171	H-2->L+1 (43%), H-1->LUMO (43%)	H-2->LUMO (5%), H-1->L+1 (5%)

Electronic transitions in Cd^0@2ML-GQDs

No.	Wavelength (nm)	Osc. Strength	Major Contribs	Minor Contribs
2	482.29	0.0287	H-1->LUMO (46%), HOMO->L+1 (48%)	H-3->L+2 (2%)
5	454.12	0.001	H-2->LUMO (80%), H-2->L+2 (16%)	H-1->LUMO (2%)
8	431.77	0.3586	H-1->LUMO (45%), HOMO->L+1 (40%)	H-4->L+3 (3%), H-3->L+2 (8%), H-2->LUMO (2%)
10	412.71	0.0029	H-4->LUMO (45%), H-1->L+3 (12%), HOMO->L+2 (35%)	H-3->L+1 (3%)
11	406.73	0.0244	H-3->L+1 (46%), H-1->L+3 (35%), HOMO->L+2 (12%)	H-4->LUMO (4%)

Electronic transitions in Cd^0@3ML-GQDs

No.	Wavelength (nm)	Osc. Strength	Major Contribs	Minor Contribs
4	495.50	0.0035	H-1->L+1 (72%), HOMO->LUMO (21%)	
6	466.72	0.0489	H-4->L+1 (10%), H-3->LUMO (19%), H-1->L+3 (20%), HOMO->L+2 (43%)	H-5->L+3 (3%)
7	459.64	0.2057	H-1->LUMO (30%), HOMO->L+1 (53%)	H-5->LUMO (5%), H-5->L+4 (3%), H-3->L+3 (4%)
8	450.95	0.0061	H-2->LUMO (60%), H-2->L+3 (31%)	H-2->L+4 (6%)
9	445.69	0.0205	H-5->LUMO (52%), H-3->L+3 (11%)	H-6->L+1 (3%), H-4->L+2 (8%), H-1->LUMO (2%), H-1->L+4 (9%), HOMO->L+5 (8%)

Electronic transitions in Hg^0@1ML-GQDs

No.	Wavelength (nm)	Osc. Strength	Major Contribs
3	360.16	1.3636	H-1->LUMO (49%), HOMO->L+1 (49%)
4	360.16	1.3635	H-1->L+1 (49%), HOMO->LUMO (49%)

Electronic transitions in Hg^0@2ML-GQDs

No.	Wavelength (nm)	Osc. Strength	Major Contribs	Minor Contribs
2	482.46	0.0287	H-1->LUMO (46%), HOMO->L+1 (49%)	H-2->L+2 (2%)
6	431.92	0.3625	H-1->LUMO (47%), HOMO->L+1 (40%)	H-3->L+3 (3%), H-2->L+2 (8%)
8	412.86	0.0028	H-3->LUMO (45%), H-1->L+3 (12%), HOMO->L+2 (35%)	H-2->L+1 (3%)
9	406.87	0.0246	H-2->L+1(46%) H-1->L+3(36%),HOMO->L+2 (12%)	H-3->LUMO (4%)

Electronic transitions in Hg^0@3ML-GQDs

No.	Wavelength (nm)	Osc. Strength	Major contribs	Minor contribs
4	495.46	0.0036	H-1->L+1 (73%), HOMO->LUMO (21%)	
6	466.82	0.0483	H-3->L+1 (10%), H-2->LUMO (19%), H-1->L+3 (20%), HOMO->L+2 (42%)	H-4->L+3 (3%)
7	459.45	0.2116	H-1->LUMO (31%), HOMO->L+1 (54%)	H-4->LUMO (5%), H-4->L+4 (4%), H-2->L+3 (4%)
8	445.92	0.0214	H-4->LUMO (52%), H-2->L+3 (11%)	H-5->L+1 (3%), H-3->L+2 (9%), H-1->LUMO (2%), H-1->L+4 (9%), HOMO->L+5 (9%)

Electronic transitions in Pb^0@1ML-GQDs

No.	Wavelength (nm)	Osc. Strength	Major Contribs	Minor Contribs
3	1217.08	0.0046	HOMO->LUMO (12%), HOMO->L+1 (12%), HOMO->L+2 (23%), HOMO->L+3 (43%)	HOMO->L+5 (5%), HOMO->L+10 (3%)
4	1026.86	0.0389	HOMO->LUMO (16%), HOMO->L+1 (48%), HOMO->L+2 (32%)	HOMO->L+3 (5%)
5	688.99	0.008	HOMO->L+4 (95%)	
6	654.92	0.0014	HOMO->L+3 (20%), HOMO->L+5 (65%), HOMO->L+10 (11%)	
7	524.60	0.0151	HOMO->L+7 (80%), HOMO->L+9 (14%)	
8	504.39	0.0168	HOMO->L+5 (10%), HOMO->L+9 (47%), HOMO->L+10 (28%)	HOMO->L+6 (6%), HOMO->L+7 (5%)
9	483.48	0.0473	HOMO->L+6 (65%), HOMO->L+9 (14%)	H-1->LUMO (3%), HOMO->L+7 (9%), HOMO->L+10 (4%)
10	474.14	0.0301	HOMO->L+6 (24%), HOMO->L+9 (16%), HOMO->L+10 (48%)	HOMO->L+5 (4%), HOMO->L+8 (3%)
11	467.23	0.0149	H-2->LUMO (32%), H-2->L+1 (13%), H-1->LUMO (33%), H-1->L+1 (15%)	
12	445.50	0.0213	H-2->LUMO (16%), H-1->LUMO (32%), H-1->L+1 (11%), HOMO->L+8 (34%)	H-2->L+1 (3%)

Electronic transitions in Pb^0@2ML-GQDs

No.	Wavelength (nm)	Osc. Strength	Major Contribs	Minor Contribs
3	1548.64	0.0014	HOMO->LUMO (13%), HOMO->L+1 (31%), HOMO->L+4 (13%), HOMO->L+5 (27%)	HOMO->L+2 (7%), HOMO->L+9 (5%)
4	1296.63	0.0308	HOMO->LUMO (46%), HOMO->L+1 (43%)	HOMO->L+2 (3%), HOMO->L+3 (3%), HOMO->L+4 (6%)
5	955.41	0.005	HOMO->L+3 (72%)	HOMO->L+1 (6%), HOMO->L+2 (9%), HOMO->L+4 (4%), HOMO->L+5 (5%)
7	728.71	0.0045	HOMO->L+7 (86%)	HOMO->L+6 (9%)
8	706.54	0.0014	HOMO->L+5 (25%), HOMO->L+9 (49%)	HOMO->L+10 (2%), HOMO->L+12 (5%), HOMO->L+14 (3%), HOMO->L+15 (3%), HOMO->L+16 (3%), HOMO->L+19 (4%)
9	572.09	0.0079	HOMO->L+6 (85%), HOMO->L+7 (10%)	

10	556.95	0.0078	HOMO->L+8 (22%), HOMO->L+11 (28%), HOMO->L+12 (19%), HOMO->L+13 (11%)	HOMO->L+14 (3%), HOMO->L+16 (3%), HOMO->L+17 (6%)
11	546.11	0.0133	HOMO->L+8 (57%), HOMO->L+11 (17%)	HOMO->L+9 (4%), HOMO->L+12 (6%), HOMO->L+14 (3%), HOMO->L+15 (2%), HOMO->L+16 (4%), HOMO->L+18 (3%)
12	534.29	0.0117	HOMO->L+8 (18%), HOMO->L+13 (27%), HOMO->L+16 (11%), HOMO->L+18 (10%)	HOMO->L+9 (9%), HOMO->L+11 (3%), HOMO->L+15 (8%), HOMO->L+17 (7%)

Electronic transitions in Pb^0@3ML-GQDs

No.	Wavelength (nm)	Osc. Strength	Major Contribs	Minor Contribs
4	1361.86	0.0276	HOMO->LUMO (83%)	HOMO->L+3 (8%), HOMO->L+4 (5%), HOMO->L+5 (3%)
5	1047.87	0.0069	HOMO->LUMO (10%), HOMO->L+3 (52%), HOMO->L+5 (20%)	HOMO->L+1 (7%), HOMO->L+7 (6%)
7	814.02	0.001	HOMO->L+3 (21%), HOMO->L+4 (20%), HOMO->L+5 (54%)	
9	735.15	0.0029	HOMO->L+9 (77%)	HOMO->L+4 (2%), HOMO->L+6 (3%), HOMO->L+8 (5%), HOMO->L+11 (2%), HOMO->L+12 (7%)
10	712.02	0.0014	HOMO->L+7 (25%), HOMO->L+13 (48%)	HOMO->L+16 (3%), HOMO->L+18 (5%), HOMO->L+25 (7%)
11	570.06	0.0051	HOMO->L+8 (75%)	HOMO->L+9 (6%), HOMO->L+10 (7%), HOMO->L+14 (3%)
12	559.04	0.0093	HOMO->L+16 (29%), HOMO->L+17 (16%), HOMO->L+20 (19%)	HOMO->L+8 (4%), HOMO->L+11 (6%), HOMO->L+12 (5%), HOMO->L+13 (2%), HOMO->L+15 (3%), HOMO->L+24 (5%), HOMO->L+26 (2%)

Dataset S3. Electronic transitions in HMs@GQDs: TDM analysis

Electronic transitions in Cd^0@1ML-GQDs

No.	Wavelength (nm)	Δr (A)	Integral of Overlap of Hole-electron (S)	Distance between Centroid of Hole and Electron (D, A)	Property
5	360.54	0.005886	0.7916685	0.005135	LE
6	360.54	0.006188	0.7916893	0.005133	LE

Electronic transitions in Cd^0@2ML-GQDs

No.	Wavelength (nm)	Δr (A)	Integral of overlap of hole-electron (S)	Distance between centroid of hole and electron (D, A)	Property
2	482.29	0.055313	0.7574388	0.024560	CT-LE
5	454.12	4.542171	0.0410284	3.599711	CT
8	431.77	0.153129	0.7753077	0.013221	CT-LE
10	412.71	0.031941	0.5433236	0.016546	CT-LE
11	406.73	0.070301	0.5644089	0.035899	LE

Electronic transitions in Cd^0@3ML-GQDs

No.	Wavelength (nm)	Δr (A)	Integral of Overlap of Hole-electron (S)	Distance between Centroid of Hole and Electron (D, A)	Property
4	495.50	0.137682	0.5345839	0.135600	CT-LE
6	466.72	0.768439	0.6837267	0.371514	CT-LE
7	459.64	0.142739	0.7104378	0.175389	CT-LE
8	450.95	6.210046	0.0396788	3.688614	CT-LE
9	445.69	0.164961	0.6576237	0.082279	CT-LE

Electronic transitions in Hg^0@1ML-GQDs

No.	Wavelength (nm)	Δr (A)	Integral of Overlap of Hole-electron (S)	Distance between Centroid of Hole and Electron (D, A)	Property
3	360.16	0.003237	0.7937426	0.003191	LE
4	360.16	0.003196	0.7937265	0.003191	LE

Electronic transitions in Hg^0@2ML-GQDs

No.	Wavelength (nm)	Δr (A)	Integral of Overlap of Hole-Electron (S)	Distance between Centroid of Hole and Electron (D, A)	Property
2	482.46	0.015011	0.7638382	0.004830	CT-LE
6	431.92	0.016578	0.7764734	0.005550	CT-LE
8	412.86	0.018269	0.5504031	0.005286	CT-LE
9	406.87	0.041057	0.5721309	0.015781	CT-LE

Electronic transitions in Hg^0@3ML-GQDs

No.	Wavelength (nm)	Δr (A)	Integral of overlap of hole-electron (S)	Distance between centroid of hole and electron (D, A)	Property
4	495.46	0.089563	0.5391020	0.087224	CT-LE
6	466.82	0.763005	0.6859582	0.358126	CT-LE
7	459.45	0.098694	0.7229942	0.149968	CT-LE
8	445.92	0.167817	0.6604164	0.078364	CT-LE

Electronic transitions in Pb^0@1ML-GQDs

No.	Wavelength (nm)	Δr (A)	Integral of Overlap of Hole-electron (S)	Distance between Centroid of Hole and Electron (D, A)	Property
3	1217.08	1.326988	0.2767538	1.439947	CT
4	1026.86	1.464669	0.3093754	1.792427	CT
5	688.99	2.235758	0.1690021	2.234507	CT
6	654.92	1.908978	0.2401340	1.742668	CT
7	524.60	1.888906	0.2428415	1.907628	CT
8	504.39	1.779167	0.2360076	2.011394	CT
9	483.48	2.150265	0.2016477	2.025878	CT
10	474.14	1.697126	0.1898174	2.256412	CT
11	467.23	1.043681	0.5098611	0.996061	CT
12	445.50	1.589199	0.5889999	0.294832	CT

Electronic transitions in Pb^0@2ML-GQDs

No.	Wavelength (nm)	Δr (A)	Integral of Overlap of Hole-electron (S)	Distance between Centroid of Hole and electron (D, A)	Property
3	1548.64	2.695288	0.2076104	2.153923	CT
4	1296.63	3.264488	0.2584660	2.871346	CT
5	955.41	3.550788	0.1226676	4.890277	CT
7	728.71	2.778359	0.1509912	2.341221	CT
8	706.54	2.493876	0.2050111	2.020121	CT
9	572.09	4.533741	0.0650106	4.991606	CT
10	556.95	4.234504	0.1757146	2.978327	CT
11	546.11	4.856630	0.1591862	4.416527	CT
12	534.29	4.232788	0.1740539	3.213463	CT

Electronic transitions in Pb^0@3ML-GQDs

No.	Wavelength (nm)	Δr (A)	Integral of Overlap of Hole-Electron (S)	Distance between Centroid of Hole and Electron (D, A)	Property
4	1361.86	5.040987	0.2144009	3.785214	CT
5	1047.87	4.660487	0.1382373	5.314097	CT
7	814.02	4.431046	0.0239178	7.326400	CT
9	735.15	3.706079	0.1334669	2.685261	CT
10	712.02	2.589220	0.1607910	2.476298	CT
11	570.06	5.625329	0.0638198	4.964892	CT
12	559.04	5.129562	0.1637060	3.067539	CT
